# 

**Published:** 1893-12

**Authors:** 


### CONTENTS.

ORIGINAL ARTICLES—

Operative Treatment of Knee Joint Disease. By De Forest Willard, M. D. .... 631

The Differentiation of Acute Broncho-Pneumonia and Bronchitis in Children. By Henry E. Tuley, M. D., Louisville, Ky. .... 641

Parenchymatous Aspiration—A New Method of Diagnosis. By Albert Abrams, M. D., San Francisco, Cal. .... 645

Some Practical Hints in Testing for Albumin in Urine. By E. C. Davis, A. B. and M. D. .... 650

Small Cell Sarcoma of Nasal and Orbital Cavity, Abscesses of Orbital Cavity and Necrosis of Orbital Plate of Frontal Bone, with Operations. By A. Bethune Patterson, M. D., Atlanta, Ga. .... 652

SOCIETY REPORTS—

The Clinical Society of Maryland .... 657
Southern Surgical and Gynecological Association .... 659

EDITORIALS—

Our Board of Censors .... 674
Journal Bills .... 686
International Medical Congress .... 677
Chicago Pasteur Institute .... 677

BOOK REVIEWS—

A Treatise on the Science and Practice of Midwifery.... .... 678

SELECTIONS AND ABSTRACTS—

Local Treatment of Diphtheria.......................... 656
To Cut Short Whooping-Cough in Twenty-Four Hours........ 673
A too Common Affront to the Profession.................. 684
Sennine in Eczema and Venerial Ulcers.................... 686
Nature's Anti-Fat Remedy................................ 686

PRESCRIPTION DEPARTMENT—

Expectorant for Acute Bronchitis........................ 680

Gonorrhœa................................................ 680

Cardiac Tonic............................................ 680

Nasl Catarrh............................................. 680

For Cough................................................ 681

Chronic Rheumatism....................................... 681

Anal Fissure............................................. 681

Chronic Diarrhœa......................................... 681

SPECIAL NOTES—

Eucalypton Extract....................................... 682
Lacto-Preparata.......................................... 682
Bromidia................................................. 682
Extract of Pinus Banadensis.............................. 682
Celerina................................................. 682
Cactina Pillets........................................... 688
Peacock's Bromides....................................... 688

